# Concurrent Production of α- and β-Carotenes with Different Stoichiometries Displaying Diverse Antioxidative Activities via Lycopene Cyclases-Based Rational System

**DOI:** 10.3390/antiox11112267

**Published:** 2022-11-17

**Authors:** Hao Luo, Weiwei He, Zhuqing Dai, Zhongyuan Zhang, Yihong Bao, Dajing Li, Ping Zhu

**Affiliations:** 1 College of Forestry, Northeast Forestry University, Harbin 150040, China; 2 Institute of Agro-Product Processing, Jiangsu Academy of Agricultural Sciences, Nanjing 210014, China; 3 College of Food Science and Technology, Nanjing Agricultural University, Nanjing 210095, China; 4 Jiangsu Key Laboratory for Food Quality and Safety-State Key Laboratory Cultivation Base, Ministry of Science and Technology, Nanjing 210014, China

**Keywords:** α/β-carotene, lycopene cyclase, carotenoid biosynthesis, antioxidant activity, rational system

## Abstract

α- and β-carotenes belong to the most essential carotenoids in the human body and display remarkable pharmacological value for health due to their beneficial antioxidant activities. Distinct high α-/β-carotene stoichiometries have gained increasing attention for their effective preventions of Alzheimer’s disease, cardiovascular disease, and cancer. However, it is extremely difficult to obtain α-carotene in nature, impeding the accumulations of high α-/β-carotene stoichiometries and excavation of their antioxidant activities. Herein, we developed a dynamically operable strategy based on lycopene cyclases (LCYB and LCYE) for concurrently enriching α- and β-carotenes along with high stoichiometries in *E. coli*. Membrane-targeted and promoter-centered approaches were firstly implemented to spatially enhance catalytic efficiency and temporally boost expression of TeLCYE to address its low competitivity at the starting stage. Dynamically temperature-dependent regulation of TeLCYE and TeLCYB was then performed to finally achieve α-/β-carotene stoichiometries of 4.71 at 37 °C, 1.65 at 30 °C, and 1.06 at 25 °C, respectively. In the meantime, these α-/β-carotene ratios were confirmed to result in diverse antioxidative activities. According to our knowledge, this is the first time that both the widest range and antioxidant activities of high α/β-carotene stoichiometries were reported in any organism. Our work provides attractive potentials for obtaining natural products with competitivity and a new insight on the protective potentials of α-/β-carotenes with high ratios for health supply.

## 1. Introduction

Carotenes, as a valuable class of dietary carotenoids, have been increasingly appealing due to their beneficial antioxidant activities in health care [1,2]. α-/β-Carotenes are the major isomers of carotenes and have been identified as the most essential carotenoids in the human body, together with lycopene, lutein, and β-cryptoxanthin [3,4]. More attention has been paid to the roles of β-carotene supply, owing to its much higher levels in natural products than α-carotene [5]. Recently, α-carotene is becoming more attractive, as a higher intake of dietary α-carotene is associated with lower risks of Alzheimer’s disease, cardiovascular disease (CVD), and cancer [6,7,8]. In addition, α-carotene intake is potentially of high pharmacological value in weight health, eye health, and bone health [9,10,11]. Beyond the framework of dietary intake, detailed serum concentration studies have proved that CVD requires more α-carotene serum than cancer to prevent death, indicating that different types of diseases require distinct α-carotene contents with desirable protective potentials [12,13,14]. From a health and wellness perspective, various high contents of α-carotene are cornerstones in clinical treatments [15]. Due to the remarkable negative effect on α-carotene uptake by increasing contents of β-carotene [3], various high α-/β-carotene ratios become essential to facilitate their distinct protective potentials. Generally, the protective potentials of α-/β-carotenes are closely associated with their antioxidant capacities, suggesting that α-/β-carotene ratios could be expected to possess different antioxidative activities. However, there are few reports about the antioxidative activities of α-/β-carotene ratios that explore their relationship with clinical treatments. Therefore, it is crucial to achieve various high stoichiometries of α-carotene concurrently with β-carotene to advance the knowledge of their antioxidant capacities, as well as to meet the great demand in health supply chains.

Although α-carotene and β-carotene are widely distributed in plants (vegetables, fruits, flowers, etc.), their average proportions are generally low in total carotenoids for efficient isolations of both α-/β-carotenes [16,17]. Orange, carrot, and banana are the particular species with relatively high α-carotene proportions, whereas their α-/β-carotene stoichiometries are extremely low and season-dependent [18,19]. Additionally, the outputs of α-carotene and β-carotene are severely limited due to low content, slow growth, and large land requirement of plants. To achieve higher yield, microbial production of α-carotene and β-carotene is a sustainable and feasible alternative to meet the health-supply demand, as it has the various advantages of an easy scale-up process, high productivity, and climate-independent pattern [20,21,22]. Previously, α-carotene and β-carotene have been successfully produced with high yields in *Escherichia coli* and *Corynebacterium glutamicum* by static engineering strategies [23,24]. Unfortunately, only one carotene or constant α-/β-carotene stoichiometry was achieved. Considering the distinct demands for clinical treatments, we speculated to obtain parallel productions of α-/β-carotenes along with distinct high α-/β-carotene stoichiometries by a dynamic regulation system in a genetically tractable *Escherichia coli* host. 

**Figure 1:**
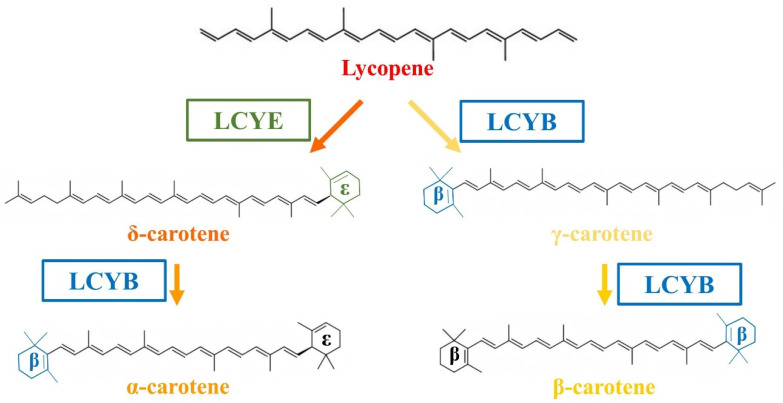
Biosynthesis pathway for α-carotene and β-carotene. Red arrows represent the α-carotene-forming branch, while black arrows represent competing branch leading to β-carotene. Enzymatic conversions were shown by arrows with related enzymes involved in each reaction. The conversion of lycopene to β-carotene requires only lycopene β cyclase. The conversion of lycopene to α-carotene requires both lycopene β and ε cyclases.

The productions of α-carotene and β-carotene originate from a lycopene asymmetric cyclization process (Figure 1) [25]. High competitivity of lycopene β-cyclase (LCYB can simultaneously cyclize both ends of lycopene) against lycopene ε-cyclase (LCYE performs one end cyclization) diverts the main flux into β-carotene production instead of α-carotene, indicating an initial requirement to properly balance the cyclization orders of LCYB and LCYE to obtain both products. As the substrate lycopene is lipophilic [26] and accumulated in a membrane, spatial separation of LCYB and LCYE would be a straightforward approach to address cyclization orders. *E. coli* localizing tags have been successfully used to target various proteins to different cell compartments for optimizing protein locations [27], while the tags have not been reported to spatially localize LCYB and LCYE. In addition, shutting down the initial expression of β-cyclase by temporal control was proved to promote the formation of α-carotene in *S. cerevisiae* [28]. Thus, temporal organization of LCYB and LCYE could enhance cyclization efficiency. Recently, high stoichiometry of α-/β-carotene in plants has been reported to be related to temperature influence [29]. Herein, temperature regulation could provide a potential method to acquire distinct high α-/β-carotene stoichiometries.

In this work, we reported the efficient concurrent accumulations of α- and β-carotenes along with various high α-/β-carotene stoichiometries in *E. coli* by a lycopene cyclase-based dynamic regulation system combining the spatial, temporal, and temperature editorial strategies. In this context, we used genetic *E. coli* membranes as the sources of various high ratios of α-/β-carotene and investigated the antioxidative activities of different α-/β-carotene ratios.

## 2. Materials and Methods

### 2.1. Strains, Media, and Reagents 

*Escherichia coli* DH5α (Millipore Corp., Burlington, MA, USA), BL21 (DE3) (Millipore Corp., Burlington, MA, USA), and XL-1 Blue (Agilent Technologies, Waldbronn, Germany) strains were used in this study. The shake flask fermentation was performed in LB medium containing 50 μg/mL kanamycin, 100 μg/mL ampicillin, or 34 μg/mL chloramphenicol for plasmid selection and maintenance.

All chemicals used in this study were reagent grade, and purchased from Aladdin Industrial Inc. (Shanghai, China) or Sangon Biotech (Shanghai, China). All enzymes for molecular biology experiments were purchased from TaKaRa Biotech (Dalian, China), New England Biolabs (Ipswich, MA, USA), or Vazyme Biotech (Nanjing, China).

### 2.2. Plasmid Constructions for TeLCYE and TeLCYB

The codon-optimized full-length sequence of *Tagetes erecta* lycopene ε-cyclase (TeLCYE, UniProt ID: Q8L8H4) was synthesized into pET28a vector by Sangon Biotech (Shanghai, China), named pET-*TeLCYE*. Then, *TeLCYE* gene with introduced C-terminal 6×His tag was amplified using primer pair TeLCYE-F/TeLCYE-R from the template plasmid pET-*TeLCYE*. The coding fragment for GlpF was amplified using the primer pair GlpF-F/GlpF-R from *E. coli* JM109 genomic DNA extracted by TIANamp Bacteria DNA Kit from TIANGEN biotech (Beijing, China) according to the manufacturer’s protocols. The *GlpF* and *TeLCYE-6×His* genes were then assembled together into high-copy pBlueScript II vector (Agilent) backbone amplified by the primer pair pBlueScript-F/pBlueScript-R by ClonExpress Ultra one-step cloning kit (Vazyme Biotech, Nanjing, China) following the manufacturer’s protocols, to generate pBlue-*GlpF*-*TeLCYE*. The resulting plasmid was then used as the template for knocking out *GlpF* by the primer pair ΔGlpF-F/ΔGlpF-R to generate pBlue-*TeLCYE*.

The codon-optimized full-length sequence of marigold (*Tagetes erecta*) lycopene β-cyclase (TeLCYB, UniProt ID: Q8L8H5) was synthesized into pET28a vector by Sangon Biotech (Shanghai, China) to generate pET-TeLCYB. The resulting plasmid was then used as the template for amplifying pET-ΔT7-TeLCYB backbone fragment by the primer pair pET-F/pET-R. The CspA promoter, CspA 5’UTR region, and *lac* operator sequences were amplified from pCold-TF vector (TaKaRa, China) following the reported method [30]. The resulting CspA sequence was then inserted into the original T7 promoter region of pET-TeLCYB backbone by ClonExpress Ultra One Step Cloning Kit (Vazyme Biotech, Nanjing, China), to generate plasmid pCspA-TeLCYB. All used primer pairs in this section are listed in Table S1.

### 2.3. Protein Expression Validation of Membrane-Targeted TeLCYE

The resulting pBlue-*GlpF*-*TeLCYE* was transformed and expressed in *E. coli* BL21 (DE3) at 37 °C and 200 rpm for 48 h in 100 mL LB medium. Harvested cells by 10 min centrifugation (6000× *g*) were suspended in the lysis buffer (20 mM Tris pH 7.5, 150 mM NaCl, 1% phenylmethyl sulfonyl fluoride, and 1 mg/mL lysozyme). Cell disruption was assessed by sonication and the removal of cell debris was performed by centrifugation at the 15,000× *g* for 10 min at 4 °C. Then, the supernatant was obtained and cell membrane was collected by an ultracentrifugation (150,000× *g*, 2 h). Membrane was resuspended in solubilization buffer containing 20 mM Tris pH 7.5, 150 mM NaCl, and 2.5% n-dodecyl-β-D-maltopyranoside using a Dounce homogenizer. After 2 h solubilization, supernatant with protein/detergent was collected by ultracentrifugation at 150,000× *g* for 2 h. These resulted fractions were collected for SDS–PAGE validation.

### 2.4. Quantitative Real-Time Reverse Transcription PCR

Total RNA was extracted by PureLink RNA Mini Kit (Thermo Fisher Scientific, Waltham, MA, USA) following the manufacturer’s protocols. Quantitative real-time PCR of cDNA was performed by the PrimeScript RT Master Mix (TaKaRa, China). To examine expression of TeLCYB, cDNA sample was used as the template in quantitative real-time PCR assay by Powerup SYBR Green PCR Master Mix (Thermo Fisher Scientific, USA). The 16SRNA was used as the internal standard. The primers of targeted mRNA are listed in Table S2. Raw data of RT-PCR were investigated using Quant Studio Design & Analysis Software (Thermo Fisher Scientific). Relative expression levels were analyzed by the 2^−ΔΔCT^ method.

### 2.5. Carotenoid Quantification

The plasmid pAC-LYC (Addgene plasmid #53270), harboring *crtE*, *crtB*, and *crtI* genes, was used to provide substrate lycopene [31]. To investigate the catalytic efficiency of membrane-targeted TeLCYE, pBlue-*GlpF*-*TeLCYE* or pBlue-*TeLCYE* was separately co-transformed with pAC-LYC into XL-1 Blue strain and cultured in shake flask for 72 h at 37 °C and 200 rpm. To examine the role of CspA promoter in regulating TeLCYB expression, pCspA-TeLCYB and pAC-LYC were co-transformed into XL-1 Blue strain and cultured in 100 mL LB for 24 h at 37 °C, followed by cold shock treatment at 10 °C for 2 h. The flask was then cultivated at 37 °C until 72 h termination. To analyze the effect of CspA-LCYB and membrane-targeted LCYE on the product accumulation, pAC-LYC, pCspA-*TeLCYB,* and pBlue-*TeLCYE* were co-transformed into XL-1 Blue strain and inoculated in 100 mL LB medium for 72 h at 37 °C as the control to determine product accumulation. Then, pAC-LYC, pCspA-*TeLCYB,* and pBlue-*GlpF*-*TeLCYE* were co-transformed into XL-1 Blue strain and inoculated in 100 mL LB medium at 25 °C, 30 °C, 37 °C for 72 h with or without cold shock treatment.

Carotenoids were extracted from cell pellets by centrifugation at 8000 rpm for 5 min with an appropriate amount methanol and chloroform (2:1 *v/v*) solution. The chloroform phase was collected after centrifugation at 8000 rpm and dried with nitrogen, followed by redissolving in 200 μL of chloroform. Samples were centrifuged and subjected to HPLC analysis. The conditions for HPLC system were consisted of YMC-C30 column (4.6 mm × 250 mm, 5 μm), column temperature of 25 °C, mobile phase using methanol-methyl tert-butyl ether (MTBE, HPLC grade, Tedia, Fairfield, OH, USA)–water of 81:15:4 (*v/v/v*) and 6:90:4 (*v/v/v*) for solvent A and B, respectively, flow rate of 1.0 mL/min, injection volume of 20 μL, detection with DAD, and detection wavelength of 450 nm. The gradient elution was developed from 1% B to 100% B within 90 min.

### 2.6. Determination of Antioxidant Property

#### 2.6.1. DPPH Free Radical Scavenging Ability Assay

Cell membranes (with or without carotenes) were resuspended in 1 mL distilled water, followed by 200 times dilution. The diluted solution was mixed with an equal volume of 0.1 mol/L DPPH free radical solution. After incubating the mixed solution at 25 °C for 30 min in dark, the absorbance was recorded at 517 nm. In this study, 95% ethanol was used as blank control; result was expressed in terms of Trolox equivalent (μmol TEAC/mL) [32].

#### 2.6.2. Determination of ABTS Free Radical Scavenging Ability

Cell membranes (with or without carotenes) were resuspended in 1 mL distilled water, followed by 30 times dilution. Then, 0.1 mL of the dilution was mixed with 3.9 mL of ABTS working solution. After incubating the reaction mixture for 6 min at 25 °C in dark, the absorbance was recorded at 734 nm. Distilled water was used as blank control; results were expressed in terms of Trolox equivalent (μmol TEAC/mL) [33].

#### 2.6.3. Determination of Ferric-Reducing Antioxidant Power (FRAP)

Cell membranes (with or without carotenes) were resuspended in 1 mL distilled water, followed by 30 times dilution. Then, 2.5 mL of the diluted solution was mixed with 2.5 mL of 0.2 mmol/L phosphate buffer (pH 6.6) and 2.5 mL of 1% potassium ferricyanide solution (*w/v*), followed by water bath (50 °C) incubation for 20 min. Then, this mixture was immediately cooled and supplemented with 2.5 mL of 10% trichloroacetic acid solution (*w/v*) followed by a 10 min centrifugation at 25 °C (3000 r/min). The collected supernatant (2.5 mL) was supplied with distilled water (2.5 mL) and 0.1% ferric chloride solution (*w/v*, 0.5 mL). The absorbance was recorded at 700 nm [34].

#### 2.6.4. Determination of MDA Content

Cell membranes (with or without carotenes) were resuspended in PBS. Then, the suspension was mixed with 2.5 mL thiobarbituric acid and heated at 100 °C for 15 min, followed by quickly cooling in an ice bath. After a 10 min centrifugation (5000 r/min), the absorbance of the supernatant was measured at 450, 532, and 600 nm, respectively [35].

### 2.7. Statistical Analysis

For all experiments, three biological replicates were performed; data were expressed as means ± standard deviation. Standard statistical analyses were conducted using the GraphPad Prism 9.4 software (GraphPad Software, San Diego, CA, USA). For multiple comparisons, one-way analysis of variance (ANOVA) with Tukey test was used. For two-sample unpaired comparisons, Student’s *t*-test was used. Asterisks in the figures denote significant differences as follows: * *p* < 0.05, ** *p* < 0.01, and *** *p* < 0.001.

## 3. Results and Discussion

With the purpose of resolving the low competitivity of LCYE against LCYB, LCYE with strong catalytic activity would be beneficial for driving the flux into α-carotene. Thus, choosing an efficient LCYE is the initial step. Generally, α-carotene is an intermediate and not the ultimate target in bacteria [36]; on the contrary, α-carotene can be accumulated in some plants, suggesting LCYE from plants as the better choice. Previous studies have proven that TeLCYE from *Tagetes erecta* displays a relatively stronger activity than LCYEs from other plants and has a favorable competitive relationship with TeLCYB for massive lutein (downstream product of α-carotene) accumulation in its flower [37], indicating TeLCYE and TeLCYB could be preferentially introduced for α-/β-carotene enrichment.

### 3.1. Construction and Expression of Membrane-Targeted TeLCYE

**Figure 2:**
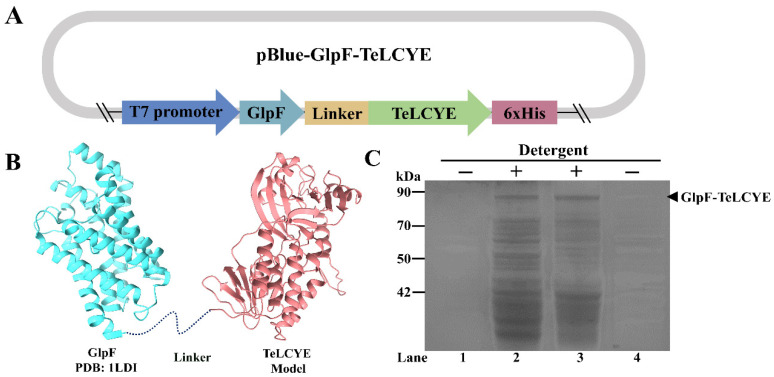
Construction and expression profiles of TeLCYE targeted to *E. coli* membrane. (**A**) The fusion structure of pBlue-*GlpF*-*TeLCYE* expression vector. GGGSGGGS was added as the linker. (**B**) The schematic model of fusion GlpF-TeLCYE. The structural model of TeLCYE was predicted by SWISS-MODEL. (**C**) SDS-PAGE validation for GlpF-TeLCYE expressed in BL21 (DE3). Lane 1, supernatant after cell lysis. Lane 2, detergent solubilized membrane supernatant after ultracentrifugation. Lane 3, detergent solubilized membrane precipitation after ultracentrifugation. Lane 4, membrane collection supernatant after ultracentrifugation.

Since the substrate lycopene is lipophilic in nature and TeLCYE is a membrane-associated enzyme with hydrophobic regions, the optimal location in the heterologous host is essential for its catalytic activity [38,39]. GlpF, as an inner membrane protein of *E. coli,* is regarded as the N-terminal fusion tag for expression of various proteins and an efficient localization tag for targeting proteins in the membrane compartment [19]. Thus, to target TeLCYE in the *E. coli* inner membrane to bring it close to its native state, it would be theoretically fused to the C-terminal of GlpF to increase the spatial accessibility to lycopene. It is well known that the introduction of a protein tag for protein expression may cause steric hindrance and greatly affect the folding performance of a protein or even inactive it. To address the steric issue in our expression system, we analyzed the structural hindrance of TeLCYE and GlpF by comparing the structural model of TeLCYE and the structure of GlpF [40]. As shown in Figure 2B, GlpF displayed a rigid C-terminal structure and TeLCYE exhibited a relatively flexible N-terminal structure. Considering the structural integrity requirement for TeLCYE activity, we sought to increase the C-terminal flexibility of GlpF by adding a flexible linker, GGGSGGGS, to its C-terminal to prevent hindrance. Moreover, a 6xHis tag was fused to the C-terminal of TeLCYE in the pBlueScript vector to validate the expression feature of the generated fusion protein (Figure 2A). After 48 h of expression at 37 °C in *E. coli*, the GlpF-TeLCYE fusion protein could barely be detected in the supernatant fraction after cell lysis (Figure 2C). Surprisingly, the fusion protein of GlpF with theoretical molecular weight (29.8 kDa) and TeLCYE with theoretical molecular weight (54.7 kDa) showed obvious molecular mass (approx. 85 kDa) in the membrane fraction after detergent solubilization, which was similar to its theoretical molecular weight (83.8 kDa). Taken together, these results preliminarily suggested that GlpF-TeLCYE fusion protein was successfully expressed and mainly targeted to the cell membrane in the *E. coli* host.

### 3.2. TeLCYE Catalytic Efficiency Enhanced via Spatially Membrane-Targeted Regulation 

**Figure 3:**
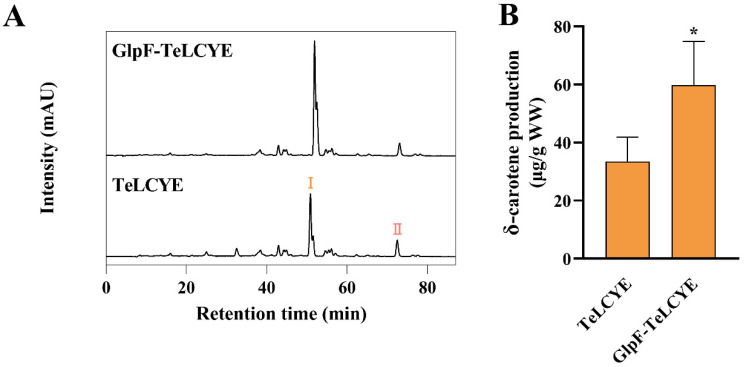
Enhanced catalytic activity of TeLCYE by membrane targeting in lycopene-accumulating *E. coli*. (**A**) HPLC spectra of XL-1 Blue strains harboring pBlue-*GlpF*-*TeLCYE* or pBlue-*TeLCYE* separately co-transformed with pAC-LYC. Ⅰ, δ-carotene; Ⅱ, lycopene. (**B**) δ-Carotene-accumulation patterns in XL-1 Blue strains harboring pBlue-*GlpF*-*TeLCYE* or pBlue-*TeLCYE* separately co-transformed with pAC-LYC. (*) *p* < 0.05.

We next attempted to consolidate the functional relevance of the membrane localization of TeLCYE in the *E. coli* host. The functional analysis of TeLCYE was investigated by transforming TeLCYE and GlpF-TeLCYE constructs into lycopene-accumulating *E. coli*, respectively. As shown in Figure 3A,B, δ-carotene as the directly cyclized metabolite was detected in both strains containing TeLCYE and GlpF-TeLCYE. It is to be noted that 1.8-fold higher δ-carotene production was obtained by membrane-targeted TeLCYE than that by free TeLCYE, indicating a strikingly improved catalytic efficiency of TeLCYE in membrane localization. In a previous study, GlpF was used as the membrane-localized tag for the expression of carotenoid cleavage dioxygenase from *Petunia hybrida* in *E. coli* without changing its function [27]. Likewise, the function of TeLCYE in our study was confirmed imperiously by GlpF in *E. coli*. In the meantime, membrane-targeted TeLCYE expressed in *E. coli* achieved the similar enhanced catalytic functionality as membrane-targeted mLCYE from *Tagetes erecta* expressed in yeast, indicating the necessity of membrane-compartment optimization for plant LCYEs expression in heterologous hosts. These results strongly confirmed that TeLCYE catalytic efficiency could be enhanced via spatially membrane-targeted regulation.

### 3.3. TeLCYB Catalytic Activity Reprogammed via Temporally Promoter-Centered Regulation

**Figure 4:**
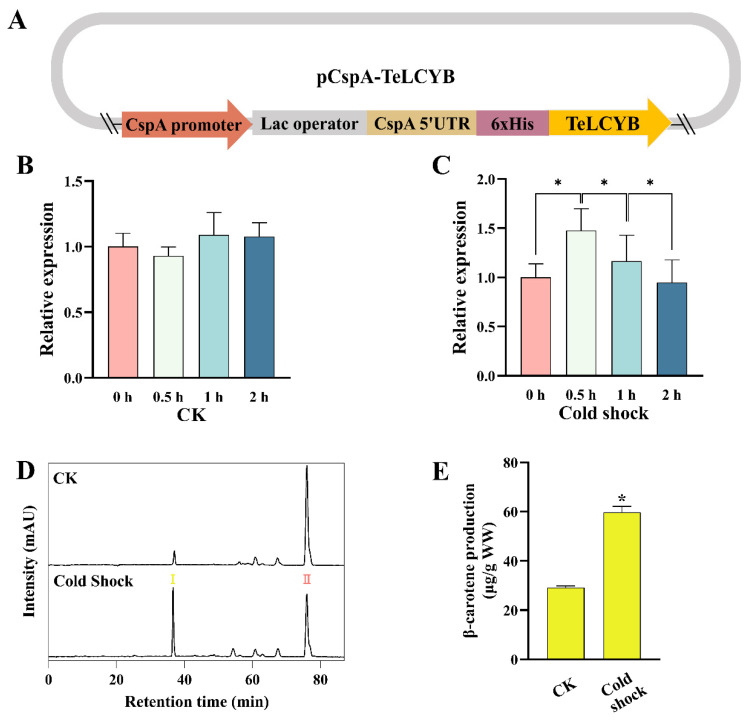
Activation of TeLCYB by CspA promoter under cold shock treatment in lycopene-accumulating *E. coli*. (**A**) The fusion structure of pCspA-TeLCYB expression vector with N-terminal CspA promoter, lac operator, CspA-5’UTR, and 6xHis tag. (**B**,**C**) The relative expression profiles of TeLCYB without (**B**) or with (**C**) cold shock treatment in two hours. (**D**) HPLC spectra of XL-1 Blue strains harboring pAC-LYC and pCspA-TeLCYB with or without cold shock treatment. Ⅰ, β-carotene; Ⅱ, lycopene. (**E**) β-Carotene-accumulation patterns in XL-1 Blue strains harboring pAC-LYC and pCspA-TeLCYB with or without cold shock treatment. (*) *p* < 0.05.

To further reinforce the competitivity of TeLCYE against TeLCYB to direct more flux into α-carotene, primarily boosted expression is crucial for TeLCYE. However, inhibiting the expression of TeLCYB at the starting stage would be a feasibly operable strategy. CspA is a cold-induced promoter of *E. coli* with a unique 5’UTR structure, unstable at 37 °C and stable after cold shock (10 °C), which inhibits gene expression at 37 °C and initiates gene expression at 10 °C [41]. Since 37 °C was applied for TeLCYE expression in our study, the inhibited expression of TeLCYB at this temperature would address the above issue. In view of these, we constructed the TeLCYB gene downstream of the CspA promoter, the lac operon, and the 5’UTR of CspA, which was then ligated into the low-copy-number pET-28a backbone without the T7 promoter (Figure 4A). To verify the expression profile of TeLCYB under the CspA promoter, the relative transcription levels of TeLCYB before and after cold shock were investigated by RT-qPCR. After 24 h of cultivation at 37 °C, strains harboring the CspA promoter and TeLCYB gene were shocked at 10 °C. As shown in Figure 4C, the TeLCYB gene maintained high expression within 2 h, with the highest up-regulated level at 0.5 h shock and back to normal expression level after 2 h, suggesting a 2 h cold shock for initiating TeLCYB expression. Notably, the transcription levels of TeLCYB gene produced few variations at 37 °C within 2 h (Figure 4B), indicating the inhibited TeLCYB expression under normal growth temperature. 

To make it clear whether the transcription level of the TeLCYB gene was responsible for the catalytic activity of TeLCYB, β-carotene contents were investigated in a lycopene-accumulating strain containing CspA-TeLCYB with or without cold shock. As shown in Figure 4D,E, β-carotene concentration was remarkably 2.04-fold increased by 2 h cold shock at 10 °C, which might be due to the high expression of TeLCYB initiated by CspA. Interestingly, a small amount of β-carotene production was achieved without cold shock treatment. Studies have shown that the CspA promoter maintains a high degree of activity at 37 °C [42], while CspA mRNA is extremely unstable at 37 °C with a short half-life and leads to a leaky expression [41]. Therefore, the small amount of β-carotene might be caused by the leaky expression of TeLCYB at 37 °C. In short, the CspA promoter system could center the temporal expression of TeLCYB for boosting its catalytic activity at the desired stage.

### 3.4. The Initially Efficient Boosted TeLCYE Is Essential for High α-/β-Carotene Stoichiometry

**Figure 5:**
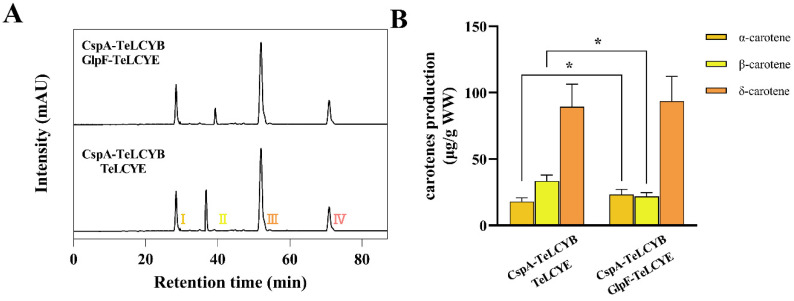
Initiation of membrane-targeted TeLCYE at the starting stage to mediate α-/β-carotene stoichiometry. (**A**) HPLC spectra of lycopene-accumulating strains harboring pBlue-*TeLCYE* or pBlue-*GlpF*-*TeLCYE* separately co-transformed with pCspA-TeLCYB cultured at 37 °C without cold shock. Ⅰ, α-carotene; Ⅱ, β-carotene; Ⅲ, δ-carotene; Ⅳ, lycopene. (**B**) Carotene-accumulation patterns in lycopene-accumulating strains harboring pBlue-*TeLCYE* or pBlue-*GlpF*-*TeLCYE* separately co-transformed with pCspA-TeLCYB cultured at 37 °C without cold shock. (*) *p* < 0.05.

In the aforementioned sections, we have proved that low competitivity of TeLCYE against TeLCYB could be addressed by the strategies of enhancing the catalytic efficiency of TeLCYE and inhibiting the catalytic activity of TeLCYB at the starting stage. To look into the desirable functions for competitive product formation, the contents of α- and β-carotenes were investigated by performing these approaches. As shown in Figure 5A,B, δ-carotene was detected as the major product for both membrane-targeted TeLCYE and free TeLCYE with inhibited TeLCYB at 37 °C, indicating high competitivity of TeLCYE by initially inhibiting TeLCYB at this stage. To be noted, the α-/β-carotene stoichiometry of 1.07 by membrane-targeted TeLCYE was 1.98 times higher than that by free TeLCYE both with inhibited TeLCYB, indicating α-/β-carotene stoichiometry was associated with the catalytic efficiency of TeLCYE at this stage. Based on these results, it could be ruled out that high α-/β-carotene stoichiometry was directly attributed to TeLCYE with high activity at the initial starting stage.

### 3.5. The High α-/β-Carotene Stoichiometries Induced by Dynamically Temperature-Programmed Regulation

**Figure 6:**
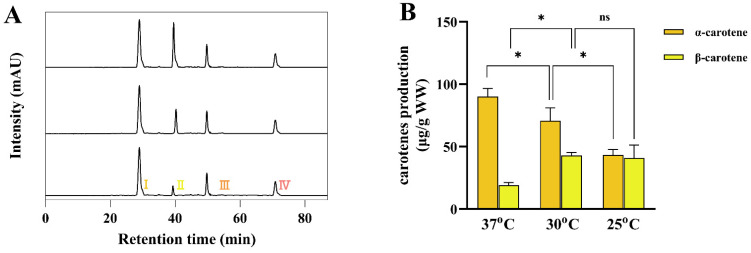
Modulation of distinct temperatures at starting stage to mediate dynamic production of α-/β-carotenes with different stoichiometries. (**A**) HPLC spectra of lycopene-accumulating strains harboring pBlue-*GlpF*-*TeLCY*E and pCspA-TeLCYB with different temperatures cultivation at starting stage and cold shock treatment at end stage. Ⅰ, α-carotene; Ⅱ, β-carotene; Ⅲ, δ-carotene; Ⅳ, lycopene. (**B**) α-/β-Carotene-accumulation patterns in lycopene-accumulating strains harboring pBlue-*GlpF*-*TeLCYE* and pCspA-*TeLCY*B with different temperatures cultivation at starting stage and cold shock treatment at end stage. (*) *p* < 0.05.

So far, the low competitivity of TeLCYE against TeLCYB was exempted at the starting stage by the above approaches; we next turned our attention to the efficient accumulation of α-/β-carotenes. Based on the above results, TeLCYE with high activity at the initial starting stage contributed the redundant δ-carotene as the major product. Thus, the efficient conversion of δ-carotene into α-carotene at the end stage became the first issue needing to be reframed. Since we assumed that TeLCYB was expected to be efficiently boosted by the CspA system at the end stage, then the obtainable amounts of α-/β-carotenes would be achieved in a rational manner. Indeed, α-/β-carotenes with a sharply decreased peak of δ-carotene were achieved in a lycopene-accumulating strain containing GlpF-TeLCYE and CspA-TeLCYB by performing the starting stage of 37 °C for 24 h and the end stage of cold shock for 2 h (Figure 6A). However, the ultimate goal was to fulfill health requirements with α-/β-carotenes along with distinct high α-/β-carotene stoichiometries at the end stage. As we have noted that α-/β-carotene stoichiometries depended on the initial activities of TeLCYE at the starting stage, the dynamical regulation of TeLCYE activity was practically essential for distinct high α-/β-carotene stoichiometries. A previous study reported that LCYE activity is temperature-dependent in *E. coli*, and increased temperature elicits stronger activity of LCYE [43]. This result encouraged us to gain insight into the effect of various temperatures on the dynamical modulating of TeLCYE activities in our study; therefore, α-/β-carotene stoichiometries were conducted by cultivating the lycopene-accumulating strain harboring GlpF-TeLCYE and CspA-TeLCYB under different temperatures at the starting stage for 24 h, followed by the same end stage of cold shock for 2 h. As shown in Figure 6B, different α/β-carotene stoichiometries finally reached 4.71 at 37 °C, 1.65 at 30 °C, and 1.06 at 25 °C, respectively, indicating the alternatively accumulated high α/β-carotene stoichiometries by performing increased temperature variations at the starting stage. These results were consistent with the primary expectation, displaying that distinct high α/β-carotene stoichiometries were easily obtained by dynamically temperature-programmed regulation.

As has been noted, high α/β-carotene stoichiometry accumulated in plants is in a specific temperature and light-dependent climate, indicating that distinct high α/β-carotene stoichiometries could be dynamically realized out of efficient operation. On the contrary, in our study, the combined strategy has displayed remarkable facility to enrich distinct high α/β-carotene stoichiometries in *E. coli*. The superior operability accumulated in *E. coli* was also exemplified by a reported study, in which the highest α/β-carotene stoichiometries from 3 to 6 were achieved by a fusion protein comprised of LCYB, LCYE, and LHC [23]. However, the potential disadvantage of this strategy is that the dynamic modulation of α/β-carotene stoichiometries could be largely limited due to the intricate genetic manipulation by gradual truncation or partial deletion of fusion proteins. In our approach, distinct high α/β-carotene stoichiometries with a wide range from 1.06 to 4.71 were performed by harnessing temperature as the convenient dynamical regulation tool. Moreover, dynamical accumulations of distinct high α/β-carotene stoichiometries in *E. coli* contributed no additional downstream products from α/β-carotenes, which were often detected in plants, and affected later extraction processes for supply. Therefore, our study provides a simple and efficient approach to facilitate the generation of substantial α/β-carotenes for health supply in *E. coli*.

### 3.6. High α-/β-Carotene Stoichiometries Contributed Distinct Antioxidant Activities

**Figure 7:**
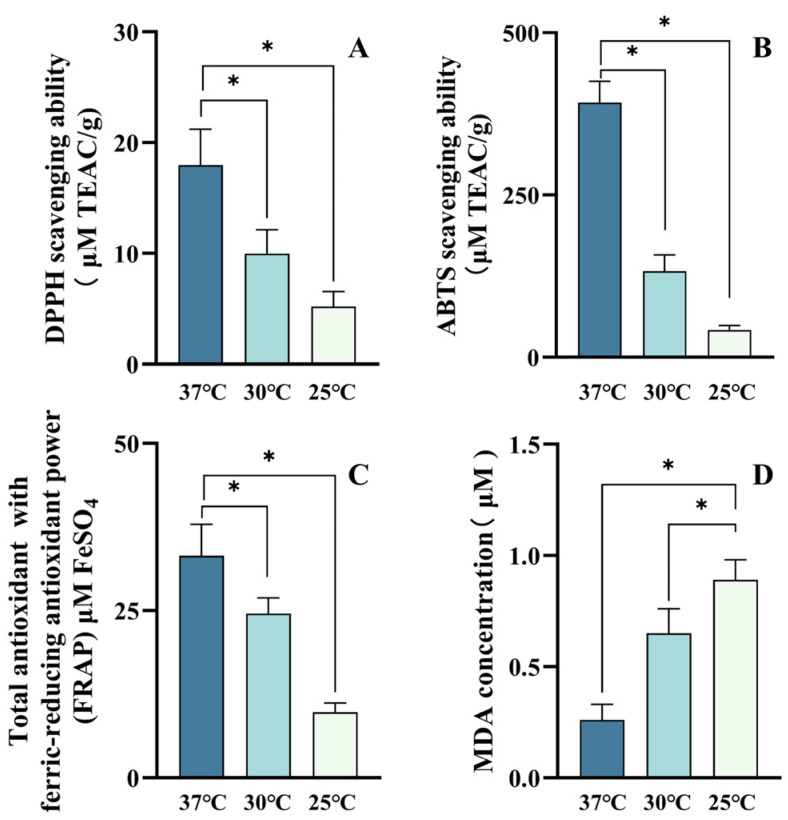
Antioxidant activities of α-/β-carotenes with different stoichiometries. DPPH scavenging ability (**A**), ABTS scavenging ability (**B**), total antioxidant with ferric-reducing antioxidant power (**C**), and MDA content (**D**) of α-/β-carotenes with different stoichiometries produced under different temperature cultivations at starting stage and cold shock treatment at end stage. (*) *p* < 0.05.

Since carotenes are lipophilic with a membrane-associated nature, the antioxidant properties of α-/β-carotenes in vitro could not be adequately presented as in situ antioxidative activities. To address this issue, their antioxidative properties were determined in a cell membrane model system based on the above genetic *E. coli* enriched with distinct high stoichiometries of α/β-carotenes. Antioxidant capacity is generally evaluated by free radicals scavenging or neutralizing potency by biological molecules; DPPH, ABTS, and FRAP are commonly used to scavenge radicals for antioxidant activity assessment [44]. As shown in Figure 7, all scavenging activity values of DPPH, ABTS, and FRAP were increased in genetic membranes containing α/β-carotenes compared with the baseline of a control membrane without α/β-carotenes. According to the reference, a genetic *E. coli* membrane containing hydroxycarotenoids achieved improved antioxidant activity [45]. In our study, enhanced antioxidant activity was displayed in a genetic *E. coli* membrane with enriched α/β-carotenes. This result confirmed that genetic *E. coli* membranes could be employed for in situ antioxidative activity determinations for α/β-carotene ratios. It is to be noted that the highest α/β-carotene ratio resulted in the highest DPPH scavenging activity value (17.97 ± 3.25 μM TEAC/g) (Figure 7A). In the meantime, distinct α/β-carotene ratios achieved different values of DPPH scavenging activity. Moreover, the highest scavenging activity values of ABTS (392.15 ± 33 μM TEAC/g) and FRAP (33.2 ± 4.7 μM FeSO_4_) were both obtained under the highest α/β-carotene ratios (Figure 7B,C). α/β-Carotene ratios contributed different values of ABTS and FRAP scavenging activities, which exhibited similar tends as those of DPPH. These results clearly indicated that the diverse antioxidative activities were related to distinct α/β-carotene ratios in genetic membranes. MDA is a key indicator reflecting cell antioxidant status, which is the main product of lipid peroxidation [46]. As shown in Figure 7D, the lowest MDA value was achieved from the membrane with the highest α/β-carotene ratio, and MDA contents increased with decreased α/β-carotene ratios. Since a lower MDA value exhibits better cell antioxidant status [47], the highest α/β-carotene ratio highly improved the antioxidant status of the genetic membrane. Thus, it could be ruled out that distinct α/β-carotene ratios contributed distinct antioxidant activities of genetic membranes.

## 4. Conclusions

In all, we reported a potentially operable and flexible strategy to enrich α/β-carotenes and uncover the dynamical regulation role of temperature in distinct high α/β-carotene stoichiometries generation in *E. coli*. The diverse antioxidative activities of distinct α/β-carotene ratios were explored using genetic *E. coli* membranes. To our delight, the widest range of high α/β-carotene stoichiometries was achieved in an *E. coli* host for the first time. Moreover, this is the first report to present antioxidant activities regarding distinct high ratios of α/β-carotenes. Our approach is of synthetic advances to enable the facile adjustment of the ratios for competitive products with important health benefits and potential applications.

## Data Availability

The data is contained within the article and supplementary.

## Supplementary Materials

The following supporting information can be downloaded at: https://www.mdpi.com/article/10.3390/antiox11112267/s1, Table S1: Primers used in this study; Table S2: Primers for qRT-PCR.
