# Supplementary material for: Concurrent Production of α- and β-Carotenes with Different Stoichiometries Displaying Diverse Antioxidative Activities via Lycopene Cyclases-Based Rational System
Source: Antioxidants (Basel). 2022 Nov 17;11(11):2267. doi: 10.3390/antiox11112267 (PMC9686992; doi:10.3390/antiox11112267)
Supplement: Supplementary file 1 [file antioxidants-11-02267-s001.zip › antioxidants-2012501-supplementary.pdf]

Supplementary Materials

Table S1. Primers used in this study

Prime name	Primer Sequence (5'-3')
GlpF-F	GTACCGGGCCCCCCCTCGAGATGAGTCAAACATCAACCTT GAAAGG
GlpF-R	TGAAGAAGATGAACCTCCCAGCGAAGCTTTTTGTTC
TeLCYE-F	GCGGATCCGGAGGCGGATCTATGTCCATGCGTGCGGGC
TeLCYE-R	TCAGTGATGATGGTGATGGTGGATGGTCAGGTACGCTTTC AGC
pBlueScript-F	CACCATCACCATCATCACTGAGGATCCACTAGTTCTAGAGC GG
pBlueScript-R	CTCGAGGGGGGGCCCGGTACCCAATTCGCCCTATAGTGAG TCGTA
ΔGlpF-F	GTACCGGGCCCCCCCTCGAGATGGTGAGCAAGGGCGAGG A
ΔGlpF-R	TCCTCGCCCTTGCTCACCATCTCGAGGGGGGGCCCGGTAC
CspA-F	AGATCTCGATCCCGCGAAATCCGATTAATCATAAATATGA
CspA-R	GTGATGATGATGATGATGCACTTTGTGATTCATGGTGTAT
pET-F	CATCATCATCATCATCACAGCAGCGG
pET-R	ATTTCGCGGGATCGAGATCTCGATCCTCTA

Table S2. Primers for qRT-PCR

Prime name	Sequence (5'-3')
16SRNA-F	CTGGTAGTCCACGCCGTAAAC
16SRNA-R	GGCACATTCTCATCTCTGAAAACTT
TeLCYB-F	TCCTGTCTAGCCGTCTGTTCCTG
TeLCYB-R	GGTGCCTTTCGCCATGATTTCAAC
